# Inhibiting Glycine Decarboxylase Suppresses Pyruvate-to-Lactate Metabolism in Lung Cancer Cells

**DOI:** 10.3389/fonc.2018.00196

**Published:** 2018-06-01

**Authors:** Chern Chiuh Woo, Kavita Kaur, Wei Xin Chan, Xing Qi Teo, Teck Hock Philip Lee

**Affiliations:** Singapore Bioimaging Consortium, Agency for Science, Technology and Research (A*STAR), Singapore, Singapore

**Keywords:** glycine decarboxylase, lung cancer, antisense oligonucleotide, carbon-13, magnetic resonance spectroscopy

## Abstract

Glycine decarboxylase (GLDC) gene is frequently upregulated in various types of cancer including lung, prostate and brain. It catabolizes glycine to yield 5,10-methylenetetrahydrofolate, an important substrate in one-carbon metabolism for nucleotide synthesis. In this study, we used exon splicing modulating steric hindrance antisense oligonucleotide (shAON) to suppress GLDC expression and investigated its effect on pyruvate metabolism *via* hyperpolarized carbon-13 magnetic resonance spectroscopy (MRS). The MRS technique allows us to study *in vivo* metabolic flux in tumor tissues with/without GLDC-shAON intervention. Here, we show that GLDC-shAON treatment is able to suppress lung cancer cell growth and tumorigenesis, both *in vitro* and *in vivo*. The carbon-13 MRS results indicated that the conversion of pyruvate into lactate in GLDC-shAON-treated tumor tissues was significantly reduced, when compared with the control groups. This observation corroborated with the reduced activity of lactate dehydrogenase and pyruvate dehydrogenase in GLDC-shAON-treated lung cancer cells and tumor tissues. Glycolysis stress test showed that extracellular acidification rate was significantly suppressed after GLDC-shAON treatment. Besides lung cancer, the antitumor effect of GLDC-shAON was also observed in brain, liver, cervical, and prostate cancer cell lines. Furthermore, it enhanced the treatment efficacy of cisplatin in lung cancer cells. Taken together, our findings illustrate that pyruvate metabolism decreases upon GLDC inhibition, thereby starving cancer cells from critical metabolic fuels.

## Introduction

In the last decade, serine–glycine biosynthesis pathway has been reported to play an important role in tumorigenesis in various types of cancer (1, 2). Phosphoglycerate dehydrogenase (PHGDH), the enzyme that catalyzes the first step in the serine–glycine biosynthesis pathway, is amplified in human melanoma and ER-negative breast cancer (3, 4). Analysis of the metabolites from culture media across NCI-60 cancer cell lines showed that glycine consumption correlates with the growth rate of highly replicating cancer cells (5). Glycine plays an important role in *de novo* purine synthesis through direct incorporation into the purine backbone (5). Glycine decarboxylase (GLDC) is one of the four proteins in the glycine cleavage system, whereby this system transfers the methyl group from glycine to tetrahydrofolate to yields 5,10-methylenetetrahydrofolate (MeTHF). MeTHF itself is an important substrate in the one-carbon metabolism for nucleotide synthesis. Since GLDC is involved in nucleotide production, it is unsurprising that tumor cells promote this gene for their growth advantage.

Glycine decarboxylase is critical to tumor-initiating cells in non-small cell lung cancer (NSCLC), and increased expression of this gene is associated with lower survivability among lung cancer patients (6). Overexpression of GLDC is able to induce oncogenic transformation in mouse fibroblast 3T3 cells (6). When GLDC is inhibited in cells with high serine hydroxymethyltransferase 2 (SHMT2) level, the accumulated glycine is converted into toxic metabolites such as methylglyoxal and aminoacetone, resulting in cell growth arrest (7). SHMT2, a mitochondrial enzyme that catalyzes the reversible conversion of serine and glycine, also plays an important role in tumorigenesis. Elevated SHMT2 expression is associated with poor prognosis in breast and lung cancer patients (8). Both SHMT1 (cytosolic isoform) and SHMT2 are direct targets of Myc, which suggests a link between the oncogene and serine metabolism (9). Inhibiting SHMT2 suppresses cell growth in liver, brain, and melanoma cancer cells (5, 7, 10). It has been recently reported that SHMT2 desuccinylation promotes tumor cell growth (11). Despite reports suggesting the therapeutic potential of inhibiting metabolic genes along the serine–glycine biosynthesis pathway, information on the metabolic responses in treated cancer cells is still lacking.

Advances in technology have accelerated the use of molecular probes to study tumor metabolism in the clinic. Radiotracer such as ^18^F-labeled fludeoxyglucose (^18^FDG) is widely used to investigate changes in tumor glycolysis, while ^18^F-choline and ^18^F-(2S,4R)4-fluoroglutamine (^18^FGln) are commonly used to study lipid and glutamine/glutamate metabolism, respectively (12, 13). Non-radioactive methods such as magnetic resonance imaging (MRI) allow the visualization of changes in tissue morphology, while magnetic resonance spectroscopy (MRS) reveals some aspects of tissue metabolism (14). For example, carbon-13 MRS can quantify hepatic lipid and glycogen contents in real time to study metabolic disorders (14). The large chemical shift differences between the organic metabolites in a carbon-13 spectrum are unique molecular signatures that enable their individual identification within a tissue voxel (12). However, the low natural abundance (1.1%) and gyromagnetic ratio of carbon-13 limit the signal-to-noise ratio, which makes it unattractive for routine clinical use. To overcome that limitation, the carbon-13 MRS signal can be amplified by more than 10,000 times by a physical process known as hyperpolarization, in which ^13^C-labeled biomolecules are inserted into a high field magnet, frozen at 1 K, irradiated with microwaves, and finally dissolved for intravenous injection (12–16). Through this advanced imaging technology, we can now study *in vivo* organ metabolism in real time without radioactivity.

Till present, there is no available GLDC inhibitor. Thus, in this study, we used steric hindrance antisense oligonucleotide (shAON) to inhibit GLDC, followed by hyperpolarized ^13^C-pyruvate MRS to measure tumor metabolism (17). Compared with scramble-shAON treatment, GLDC-shAON displayed significantly higher cytotoxicity in a primary NSCLC tumorsphere cell line (TS32), as well as other lung cancer cell lines (A549 and H226). Its tumor suppression effect was evident in a tumor xenograft mouse model. Our MRS data showed a significant decrease in lactate-to-pyruvate ratio within the tumors treated with GLDC-shAON. Subsequent biochemical analyses revealed decreases in lactate dehydrogenase (LDH) and pyruvate dehydrogenase (PDH) activities in GLDC-shAON-treated tumors. Glycolysis stress test revealed a reduction in extracellular acidification rate upon GLDC-shAON treatment. Taken together, our findings suggest that the therapeutic effect of GLDC inhibition might be attributed to the reduced capacity of lung cancer cells to use pyruvate as a metabolic fuel for energy production.

## Materials and Methods

### Cell Culture

Primary NSCLC tumorsphere cell line (TS32) is a generous gift from Dr. Bing Lim in Genome Institute of Singapore (A*STAR Singapore). TS32 cells were cultured in DMEM/F12 medium supplemented with 0.4% bovine serum albumin (BSA), 20 ng/ml epidermal growth factor (EGF) (Promega), 4 ng/ml basic fibroblast growth factor (bFGF) (Gibco), insulin–transferrin–selenium (ITS) (Gibco) supplement, 1% sodium pyruvate, 1% NEAA (non-essential amino acids), and 1% penicillin–streptomycin in non-treated Petri dish. NL-20, A549, H226, HeLa, U-87MG, and Hep3B cell lines were purchased from ATCC. PC-3 cell line is a generous gift from Dr. Nam Young Kang in the institute. NL-20 cells were maintained in DMEM/F12 medium supplemented with 4% FBS, 1.5 g/l sodium bicarbonate, 2.7 g/l glucose, 2 mM l-glutamine, 0.1 mM NEAA, 5 µg/ml insulin, 10 ng/ml EGF, 500 ng/ml hydrocortisone, and 1% penicillin–streptomycin. A549 and Hep3B cells were maintained in DMEM medium supplemented with 10% FBS and 1% penicillin–streptomycin, while H226 cells in RPMI1640 medium with 10% FBS and 1% penicillin–streptomycin. PC-3 cells were cultured in DMEM/F12 medium supplemented with 10% FBS and 1% penicillin–streptomycin. Both HeLa and U-87MG cells were maintained in MEM medium supplemented with 10% FBS and 1% penicillin–streptomycin. All cell cultures were maintained in CO_2_ incubator with 37°C humidified atmosphere and 5% CO_2_.

### Cell Transfection With shAON

The sequence of shAON is listed in Table S1 in Supplementary Material. The transfection mixture was prepared in shAON to Lipofectamine^®^ 2000 (μl) ratio of 1:2 in OPTI-MEM medium for 20 min before added into the cell culture. For TS32 cells, the transfection was done 2 h after cell seeding. For other cell lines, the transfection was done after overnight cell seeding. The cells were transfected for 3 days before used for subsequent assays. For drug combination study, the chemotherapeutic agent (cisplatin or doxorubicin, Sigma Aldrich) was added into the cell culture medium followed by transfection mixture.

### DNA Gel Visualization to Detect Exon Skipping

Polymerase chain reaction (PCR) of cDNA products was performed with primers (Table S1 in Supplementary Material) amplifying the region to cover the target exon and neighboring exon. The PCR reaction (Kapa Biosystems) was setup according to the manufacturer’s protocol. The PCR product was loaded into 1.5% agarose gel followed by 1.5 h run. The gel was then visualized with Gel Doc (Bio-Rad).

### Reverse Transcription and Quantitative Real-Time PCR

The experiment was performed according to the manufacturer’s protocol. Briefly, cell pellet was lysed with TRIzol reagent (Invitrogen). Total RNA was underwent DNase treatment (Invitrogen) and reverse transcription (Thermo Scientific) to synthesize cDNA. Quantitative real-time PCR was setup with Maxima SYBR Green qPCR Master Mix (Thermo Scientific) in StepOnePlus™ System (Applied Biosystems). The mRNA expression was calculated using 2^−ΔΔCt^ method after normalizing to endogenous control (TBP). The sequence of each primer was listed in Table S1 in Supplementary Material.

### Western Blot

Briefly, cells or tissues were lysed/homogenized in RIPA buffer followed by protein quantification with Bradford assay (Bio-Rad). The lysate was mixed with Laemmli sample buffer and loaded into 10% SDS/PAGE gel. After gel run, the protein was then electroblotted onto nitrocellulose membrane using iBlot2 (Thermo Fisher Scientific). The membrane was then blocked with 2% BSA before probed overnight with primary antibody (1:1,000 dilution). After probed with secondary antibody (1:10,000 dilution), the fluorescence signal was read using Odyssey^®^ CLx Imaging System (LI-COR Biosciences). The source of each antibody was listed in Table S2 in Supplementary Material.

### MTT and MTS Assay

Briefly, the cells were seeded into 96-well microplate followed by drug treatment. For MTT assay, the medium was replaced by MTT-added medium (0.5 mg/ml, Sigma Aldrich) followed by 3 h incubation. The medium was then replaced by DMSO to dissolve the formazan crystals to produce color for absorbance measurement (Infinite^®^ M200, Tecan) at 570 nm. For MTS assay, 20 µl of MTS solution (Promega) was added into each well followed by 4 h incubation. The absorbance was then read at 490 nm.

### Tumorsphere Assay

This assay was performed as described previously (18). Briefly, 200 cells in 0.2 ml of tumorsphere medium (DMEM/F12 medium supplemented with ITS, 20 ng/ml EGF, 10 ng/ml bFGF, 5 µg/ml insulin, and 0.4% BSA) were loaded into each well of ultra-low attachment 96-well microplate. After drug treatment, the plate was sealed with laboratory tape to avoid evaporation followed by 1-week incubation. Number of tumorsphere in each well was counted under light microscopy.

### Tumor Xenograft Mouse Model

All animal procedures were approved by Institutional Animal Care and Use Committee from A*STAR Singapore. TS32 cells were made into single cell suspension using Accutase (Sigma Aldrich). After cell count, 100 µl of 250,000 cells in serum free medium to matrigel (BD Biosciences) ratio of 1:1 was subcutaneously inoculated into the right flank of male NOD-SCID mice (Taconic) of 4–6 weeks old. Treatment was started 2 days after cell inoculation. Three treatment groups were formed: vehicle (Veh) mice were given deionized water injection, scramble-shAON (Scr) mice were given 36 mg/kg scramble-shAON, and GLDC-shAON (GLDC) mice were given 50 mg/kg GLDC-shAON. The dose for Scr mice and GLDC mice was at the same molarity because GLDC-shAON has longer sequence thus heavier. All treatments were done three times a week *via* i.p. for 7 weeks. Tumor size was measured using caliper, and the volume was calculated using the formula of volume = (width^2^ × length)/2. Starting animal size for each group was *n* = 10, however, due to accidental death and tumor ulceration (according to IACUC protocol we need to euthanize the animal immediately), the final sample sizes included in the study are vehicle (*n* = 8), Scr (*n* = 10), and GLDC (*n* = 8). At the end of the study, the tumor tissues were harvested, snap frozen, and stored at −80°C.

### Magnetic Resonance Spectroscopy

The experiment was performed as described previously (15). Mice were anesthetized with 2% isoflurane mixed with medical air and positioned in a 9.4 T preclinical MRI scanner. 40 mg of [1-^13^C]pyruvic acid (99% carbon-13-labeled, Sigma Aldrich) doped with 15 mM trityl-radical (OXO63, GE Healthcare), and 3 µl of gadoterate meglumine (10 mM, Dotarem, Guerbet) was hyperpolarized in a polarizer with 45 min of microwave irradiation. The sample was subsequently dissolved in a pressurized and heated alkaline solution containing 100 mg 1-1 ethylenediaminetetraacetic acid to yield a solution of 80 mM hyperpolarized sodium [1-^13^C]pyruvate with a polarization of 45% and physiological temperature and pH. Tumor ^13^C MR spectra were analyzed using the AMARES algorithm as implemented in the jMRUI software package. Spectra were baseline and direct-current offset corrected based on the last half of acquired points. Peaks corresponding to [1-^13^C]pyruvate and its metabolic derivatives [1-^13^C]lactate, [1-^13^C]alanine, and [1-^13^C]bicarbonate were fitted with prior knowledge assuming a Lorentzian line shape, peak frequencies, relative phases, and line widths. Quantified peak areas were plotted against time in Excel (Microsoft).

### Glycolysis Stress Test

Glycolysis stress test (Seahorse Bioscience) was used to determine the extracellular acidification rate of treated cells. The experiment was performed according to the manufacturer’s protocol. Briefly, the cells were seeded for overnight incubation followed by 3-day shAON treatment. Glycolysis stress test was carried out to the treated cells, and the measurement was done by XF24 Extracellular Flux Analyzer (Seahorse Bioscience). The measurements were normalized to the amount of cells obtained from the same plate used for the assay.

### LDH Assay

The experiment was performed according to the manufacturer’s protocol (Biovision). Briefly, cells or tissues were homogenized with LDH assay buffer. 2 µl of lysate was added into each well and topped up to 50 µl with LDH assay buffer. 50 µl of reaction mix was added into each well followed by kinetic absorbance measurement at 450 nm for 30 min (Infinite^®^ M200, Tecan). The LDH activity of each sample was calculated based on the standard graph as well as normalization to the protein amount.

### PDH Assay

The experiment was performed according to the manufacturer’s protocol (Biovision). Briefly, cells or tissues were homogenized with PDH assay buffer. 2 µl of lysate was added into each well and topped up to 50 µl with PDH assay buffer. 50 µl of reaction mix was added into each well followed by kinetic absorbance measurement at 450 nm for 60 min (Infinite^®^ M200, Tecan). The PDH activity of each sample was calculated based on the standard graph as well as normalization to the protein amount.

### Statistical Analysis

All center values in the graphs represent mean value. Statistical analysis for group comparison was assessed using unpaired Student’s *t*-test and one-way ANOVA. Significance was represented with asterisk as follow: *p < 0.05, **p < 0.01, and ***p < 0.001.

## Results

### GLDC Expression Is Upregulated in Lung Cancer

**Figure 1:**
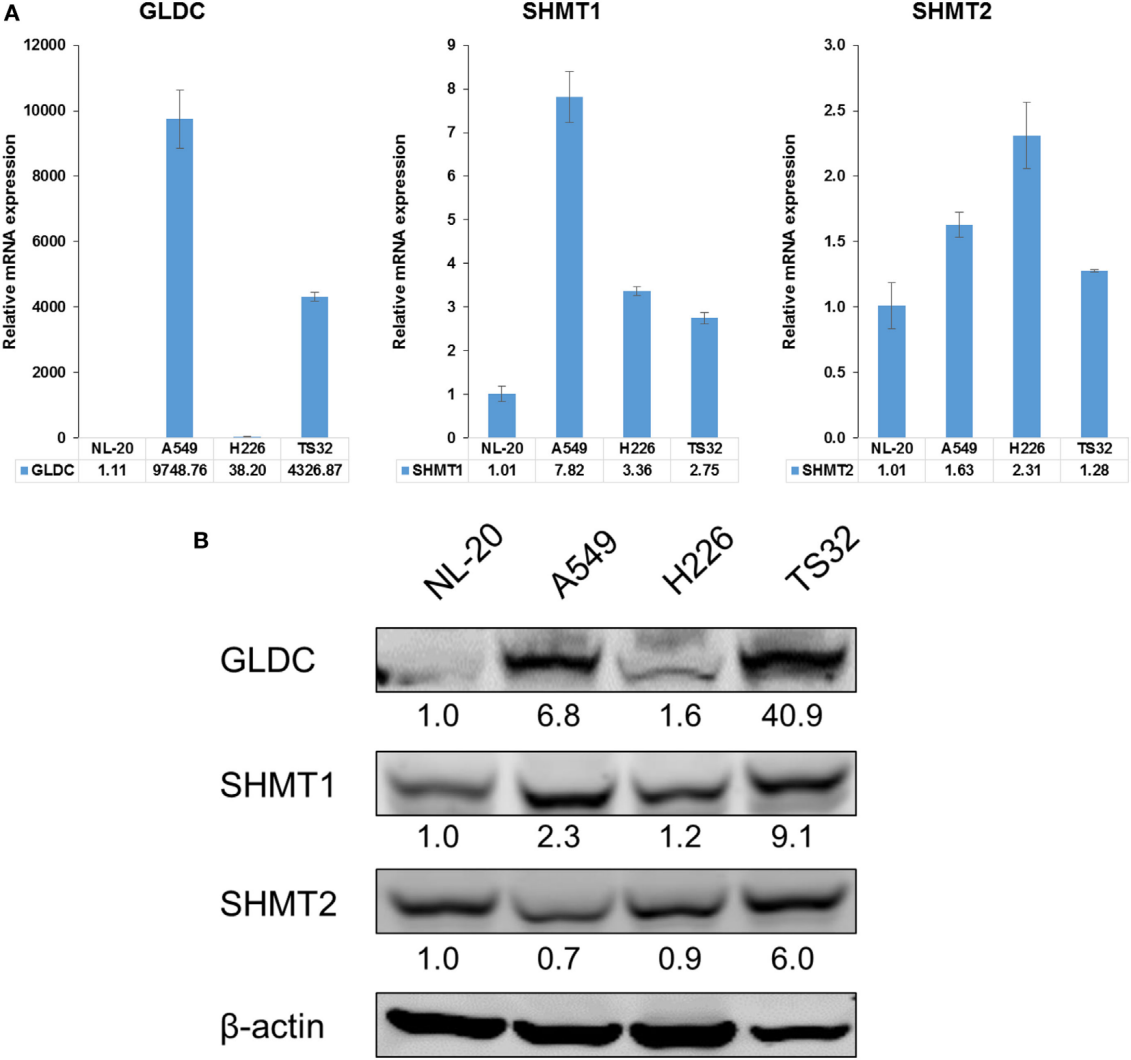
Glycine decarboxylase (GLDC) is overexpressed in lung cancer cells. **(A)** The mRNA expression of serine–glycine metabolic genes. The comparison was made between several lung cancer cell lines (A549, H226, and TS32) in relative to an immortalized normal lung cell line (NL-20). The data represent mean ± SD of triplicate polymerase chain reaction reactions. **(B)** The protein expression of serine–glycine metabolic genes in several lung cancer cell lines versus NL-20 cells. The densitometry values were in relative to NL-20 after normalization to β-actin. The data are the best representative of three independent experiments.

SHMT1/2 and GLDC are important genes in the serine–glycine metabolic pathway that are reported to be upregulated in many cancers (2). To determine whether GLDC is a potential target, we first compared its mRNA and protein expression in lung cancer cell lines against normal lung cells. In addition to commercially available lung cancer cell lines (A549 and H226), we also included a primary NSCLC tumorsphere cell line (TS32), as described in Zhang et al. (6), in the comparison (6). The mRNA (Figure 1A) and protein (Figure 1B) expression results showed that GLDC is highly expressed in these three lung cancer cell lines as compared with NL-20 lung normal cells. We also observed the increased expression of SHMT1 and SHMT2 in TS32 cells. From The Cancer Genome Atlas (TCGA), we observed that GLDC is highly upregulated in several tumor types versus its respective non-cancerous samples. As shown in Table S3 in Supplementary Material, the fold change of tumor versus normal samples of RNAseq data for cervical squamous cell carcinoma and endocervical adenocarcinoma (CESC) is 4.6, glioblastoma multiforme is 2.02, prostate adenocarcinoma is 3.81, lung adenocarcinoma (LUAD) is 2.39, lung squamous cell carcinoma is 1.59, and bladder urothelial carcinoma is 4.42. Together, these results indicate that GLDC is overexpressed in many types of cancer including lung cancer, which makes it a potential target in anticancer treatment.

### GLDC Inhibition With shAON Suppresses Lung Cancer Cells

**Figure 2:**
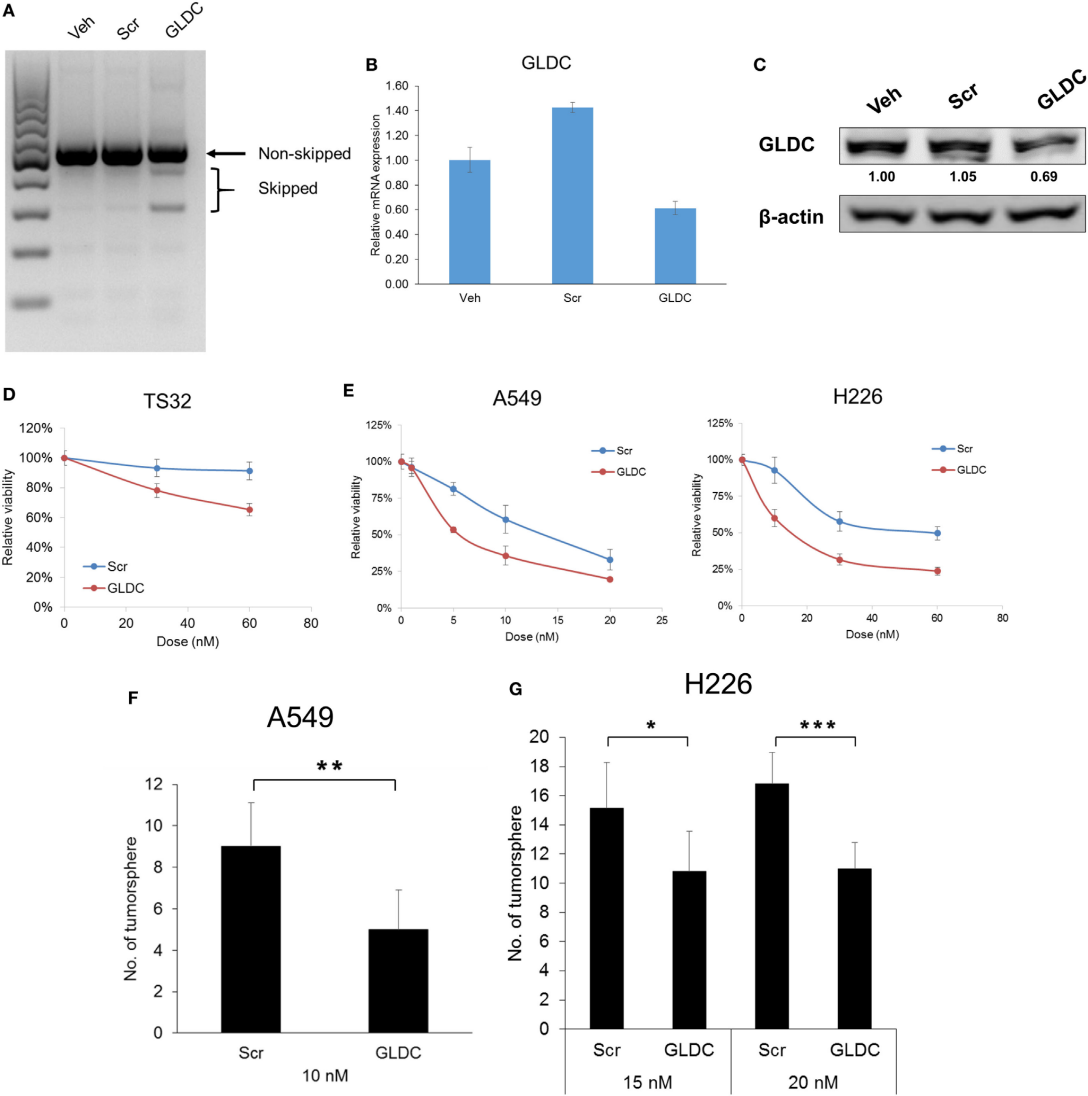
Glycine decarboxylase (GLDC) inhibition suppresses lung cancer cell growth and tumorigenicity. **(A)** Representative image of agarose gel electrophoresis of polymerase chain reaction (PCR) products. After treatment for 3 days, total RNA was harvested for cDNA synthesis followed by PCR amplification for exon skipping detection. **(B)** The mRNA expression of GLDC after steric hindrance antisense oligonucleotide (shAON) treatment. After 3-day treatment, total RNA was harvested for cDNA synthesis followed by qPCR assay. The data represent mean ± SD of triplicate PCR reactions. **(C)** The protein expression of GLDC after shAON treatment. After 3-day treatment, protein lysate was collected for western blot. The densitometry values were in relative to Veh after normalization to β-actin. The data are the best representative of three independent experiments. **(D)** MTS assay to show the effect of GLDC-shAON on cell proliferation. 3,000 TS32 cells were seeded into ultra-low attachment 96-well microplate followed by shAON transfection for 3 days. The data represent mean ± SEM of three independent experiments. **(E)** MTT assay to show the effect of GLDC-shAON on cell proliferation. 2,000 cells were seeded into 96-well microplate for overnight followed by shAON transfection for 3 days. The data represent mean ± SEM of three independent experiments. **(F,G)** Effect of shAON to tumorsphere formation in A549 and H226 cells. 200 cells were seeded into ultra-low attachment 96-well microplate followed by shAON transfection for 7 days. The data represent mean ± SD of five wells (*p < 0.05, **p < 0.01, and ***p < 0.001).

Till date, there is no GLDC inhibitor reported and available in the market. Therefore, we inhibited GLDC expression *via* the induction of exon skipping in nascent GLDC transcript (17). To study the effectiveness of this compound in suppressing lung tumorigenesis, we first treated the cells with GLDC-shAON and verified the extent of exon skipping. As shown in Figure 2A, TS32 cells treated with GLDC-shAON displayed a higher density of skipped bands (spliced GLDC transcript) and less in non-skipped band (nascent GLDC transcript) in DNA agarose gel as compared with the vehicle and scramble-shAON treatments. This observation validated the exon skipping in GLDC transcript in TS32 cells. Next, we found that both mRNA (Figure 2B) and protein expressions (Figure 2C) of GLDC were reduced after GLDC-shAON treatment but no change in vehicle and scramble-shAON treatments. We noticed that both scramble- and GLDC-shAON suppressed lung cancer cell growth, but GLDC-shAON has a more potent effect than scramble-shAON (Figures 2D,E). Moreover, we also found significantly lower number of tumorspheres in A549 (Figure 2F) and H226 cells (Figure 2G) after GLDC-shAON treatment. Together, these results suggest that GLDC-shAON-induced exon skipping suppresses GLDC expression, which resulted in reduced cell growth and tumorigenicity in lung cancer cells.

### GLDC Inhibition Suppresses Tumor Growth

**Figure 3:**
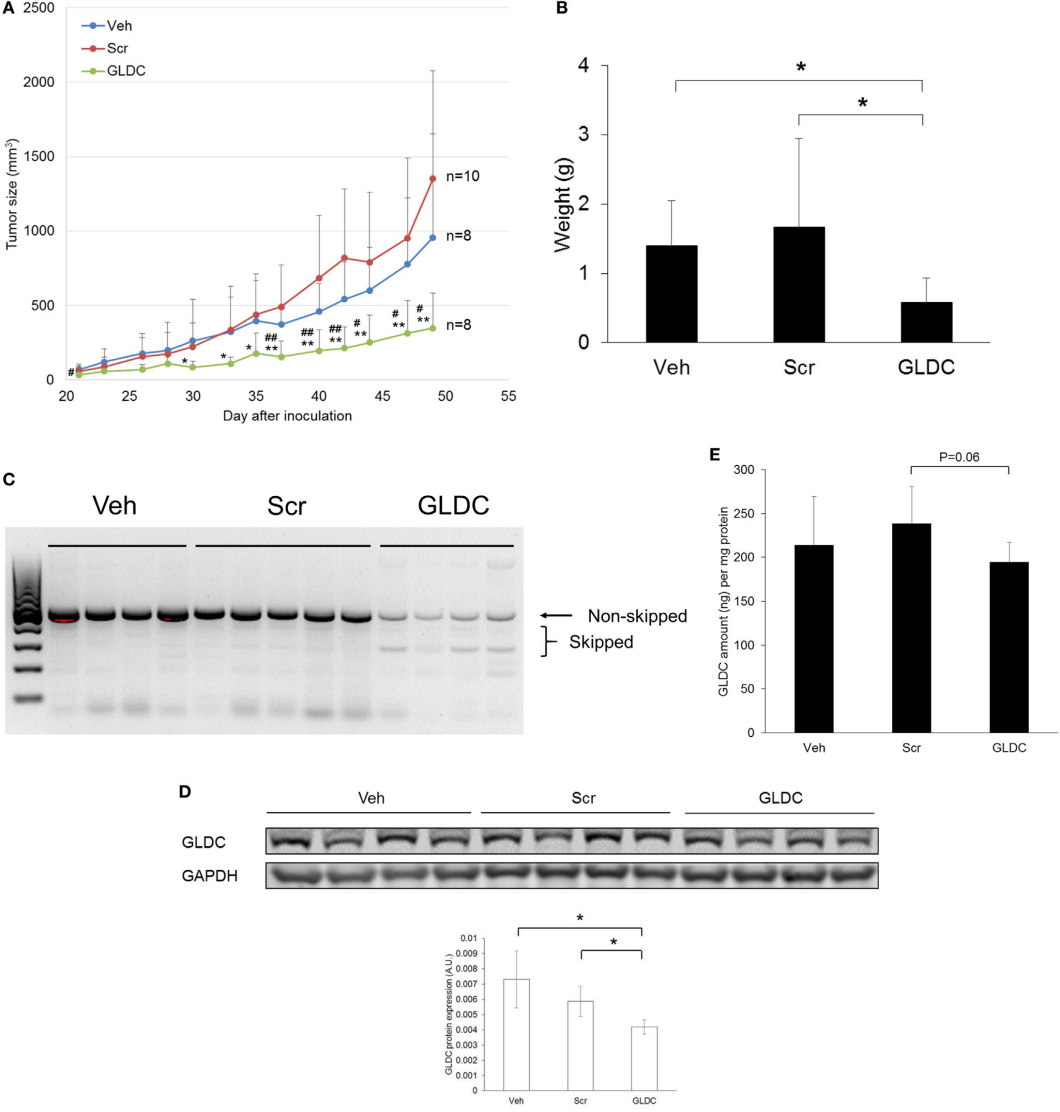
Glycine decarboxylase (GLDC) inhibition suppresses lung tumor xenograft. **(A)** Tumor growth progression of all treatment groups. TS32 cells were subcutaneously injected into the right flank of NOD-SCID mice followed by 7 weeks of treatment. Data represent mean tumor volume ± SD. GLDC versus Veh: ^#^*p* < 0.05 and ^##^*p* < 0.01. GLDC versus Scr: *p < 0.05 and **p < 0.01. **(B)** Tumor weight of all treatment groups. Tumor tissues were harvested at the end of the study for weight measurement. Data represent mean tumor weight ± SD (*p < 0.05). **(C)** Representative image of agarose gel electrophoresis of polymerase chain reaction (PCR) products for tumor tissues in all treatment groups. After tissue homogenization, total RNA was harvested for cDNA synthesis followed by PCR amplification for exon skipping detection. **(D)** The protein expression of GLDC in tumor tissues from each treatment group. The data are the best representative of three independent experiments (*p < 0.05). **(E)** GLDC ELISA assay for tumor tissues in all treatment groups. After homogenization, tissue lysates were used for the assay.

Next, we examined the therapeutic effect of GLDC-shAON in a tumor xenograft mouse model. We started treatment 2 days after cell inoculation, and three treatment groups were formed namely vehicle (Veh), scramble-shAON (Scr) and GLDC-shAON (GLDC). As shown in Figure 3A, the antitumor effect of GLDC-shAON was first observed after 4 weeks of treatment. At the end of the study, the tumor growth inhibition in GLDC group was two times greater compared with the Veh and Scr groups. We also observed the same trend in tumor weight (Figure 3B). Expectedly, exon skipping was observed in the tumor tissues of GLDC group but not in the Veh and Scr groups (Figure 3C). Similarly, western blot of the tumor tissues showed that GLDC protein expression was reduced in the GLDC group as compared with the Veh and Scr groups (Figure 3D). ELISA assay showed that GLDC protein expression in the GLDC group was lower than the Scr group albeit it was not statistically significant (*p* = 0.06) (Figure 3E). Taken together, these *in vivo* observations agree with *in vitro* results and support anti-GLDC as an effective strategy in treating lung cancer.

### [1-^13^C]Pyruvate MRS Reveals Reduced Pyruvate-to-Lactate Conversion After GLDC-shAON Treatment

**Figure 4:**
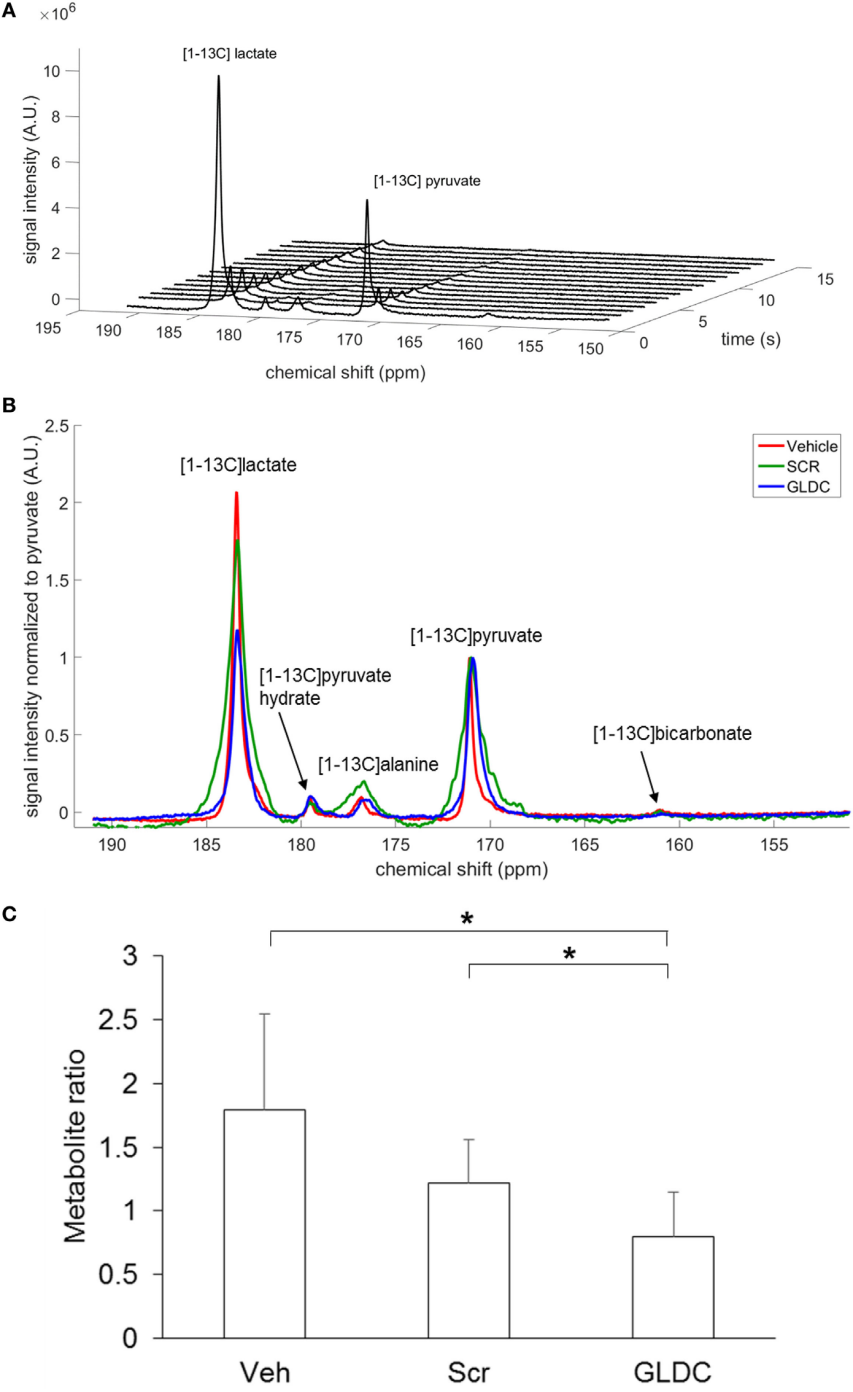
Detection of pyruvate metabolic products in the tumor using hyperpolarized ^13^C MRS. **(A)** Dynamic *in vivo* tumor ^13^C MR spectra over 1 min period after [1-^13^C]pyruvate injection, with a time resolution of 1 s. **(B)** Representative summed spectra over 10 s of all treatment groups. **(C)** Signal quantification of [1-^13^C]lactate after normalization to total carbon. Data represent mean ± SD (*p < 0.05).

To investigate the effect of GLDC inhibition in tumor metabolism, we performed [1-^13^C]pyruvate MRS to compare the pyruvate metabolism among the treatment groups. After hyperpolarization, [1-^13^C]pyruvate was immediately infused into mouse body circulation, and MRS acquisition was initiated at the tumor region for a minute over a 9.4 T scanner. A representative hyperpolarized ^13^C MR spectrum of [1-^13^C]pyruvate in tumor tissue is shown in Figure 4A. [1-^13^C]pyruvate was observed at 173 ppm while [1-^13^C]lactate at 185 ppm. A representative summed spectra over 10 s of each treatment group is shown in Figure 4B. We found that [1-^13^C]lactate level was significantly lower in the GLDC group as compared with the Veh and Scr groups (Figure 4C). These results suggest that the conversion of pyruvate-to-lactate in tumor was impaired after GLDC-shAON treatment.

### GLDC-shAON Reduces LDH and PDH Activities

**Figure 5:**
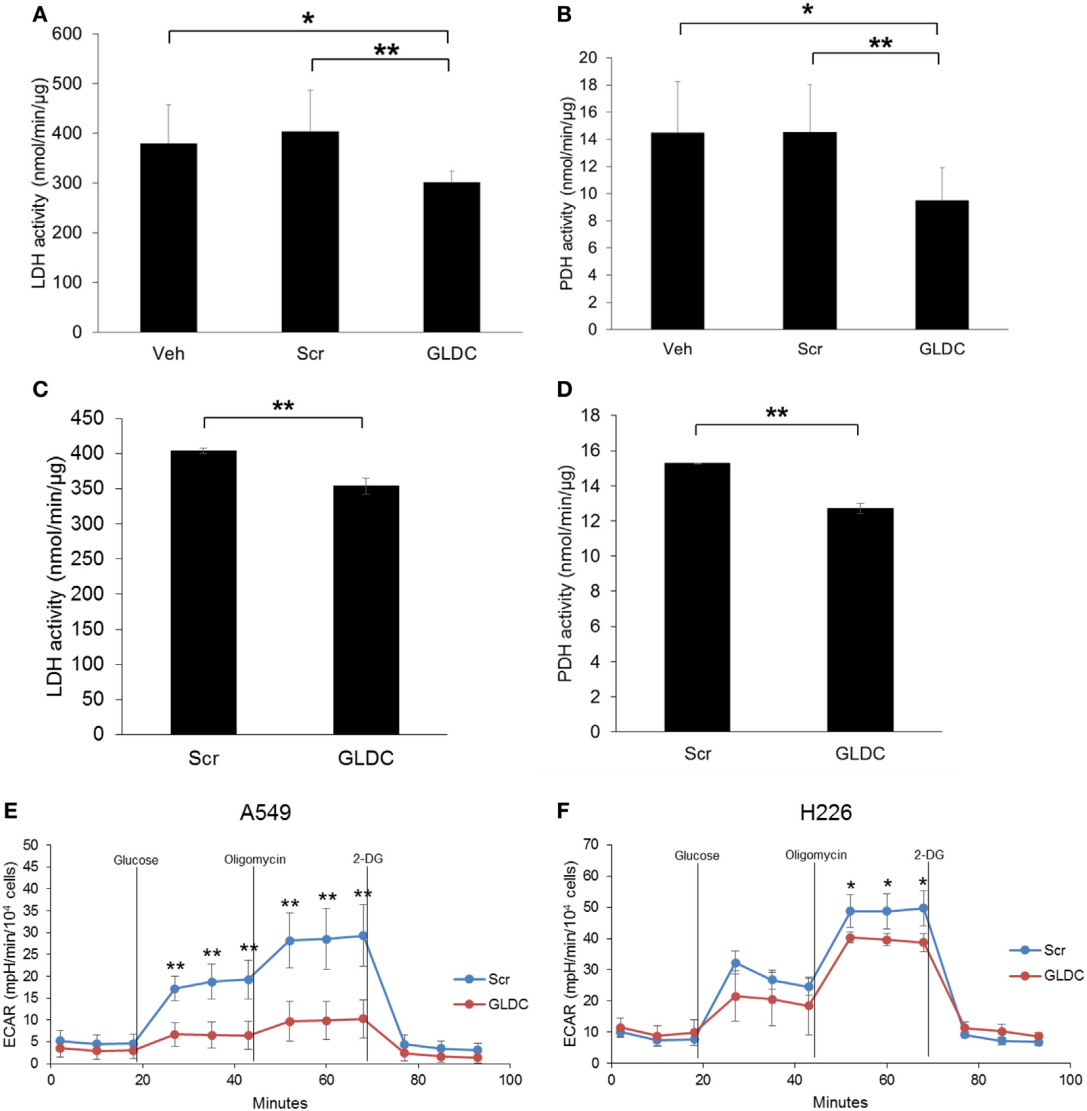
Glycine decarboxylase (GLDC)-steric hindrance antisense oligonucleotide (shAON) treatment inhibits lactate dehydrogenase (LDH) and pyruvate dehydrogenase (PDH) activities as well as reduces lactic acid production. Measurement of **(A)** LDH and **(B)** PDH activities in the tumor tissues of all treatment groups. Data represent mean ± SD (*p < 0.05 and **p < 0.01). Measurement of **(C)** LDH and **(D)** PDH activities in shAON-treated H226 and A549 cells, respectively. Data represent mean ± SD (**p < 0.01). Glycolysis stress test to measure the extracellular acidification rate in **(E)** A549 and **(F)** H226 cells after 3 days of shAON treatment. Data represent mean ± SD (*p < 0.05 and **p < 0.01).

It is widely recognized that cancer cells reprogram their metabolic pathways to sustain cell growth and survival (19). Warburg effect is a phenomenon in cancer cells where glycolysis rate is increased to metabolize more glucose to lactate even in the presence of oxygen. To verify our MRS data, we compared the LDH and PDH activities in the tumor tissues of all treatment groups. As shown in Figures 5A,B, both LDH and PDH activities were reduced in the GLDC group as compared with the Veh and Scr groups. A similar trend was also observed in lung cancer cells treated with GLDC-shAON (Figures 5C,D). In addition, we also carried out glycolysis stress test to study the capability of GLDC-shAON-treated cells in performing glycolysis. As shown in Figures 5E,F, the extracellular acidification rate in A549 and H226 cells was reduced after GLDC-shAON treatment indicating a reduction in aerobic glycolysis following GLDC inhibition. Together, these results suggest that GLDC inhibition might lead to impaired pyruvate metabolism, possibly *via* decreases in LDH and PDH activities.

### GLDC-shAON Enhances Cytotoxicity in Chemotherapy

**Figure 6:**
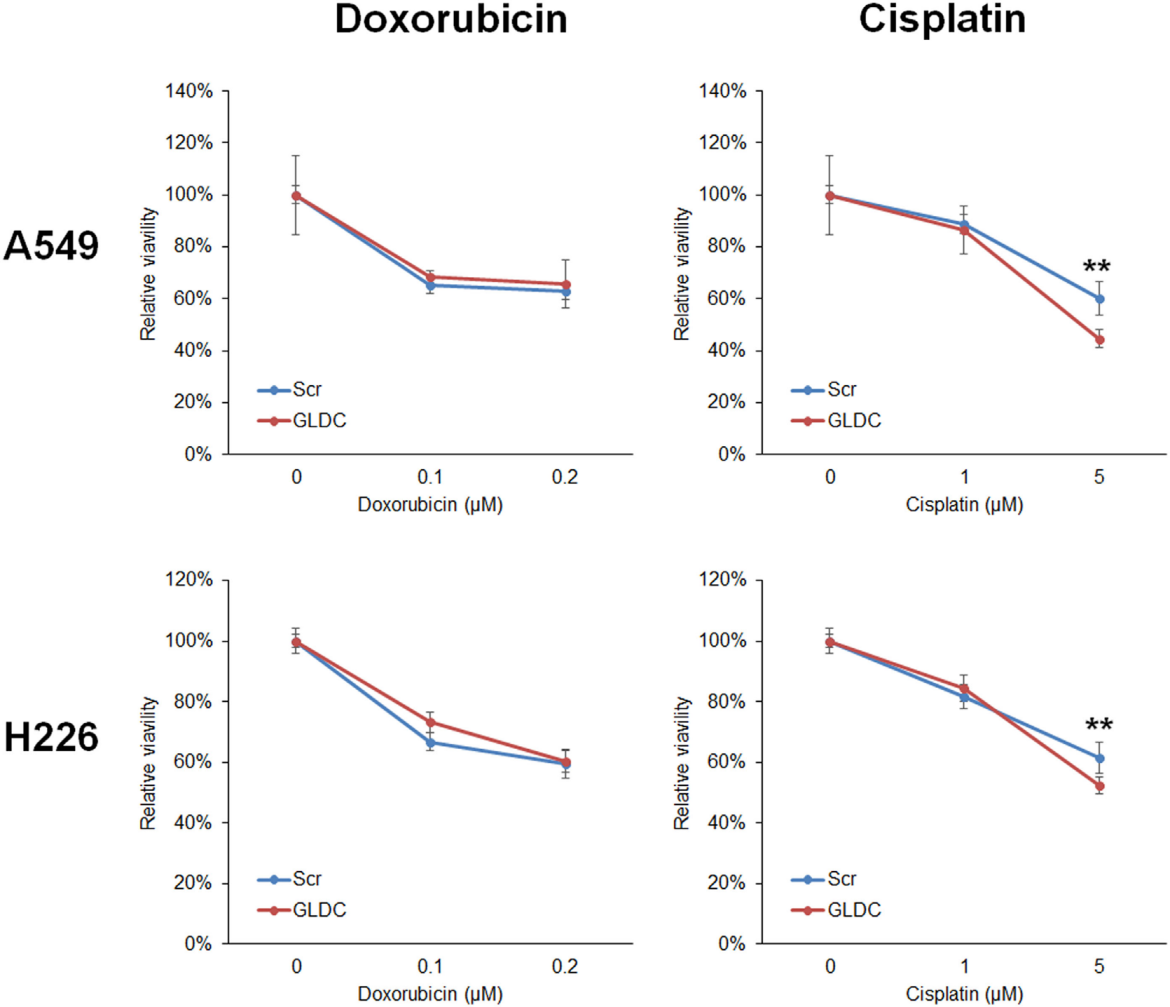
Glycine decarboxylase (GLDC)-steric hindrance antisense oligonucleotide (shAON) treatment produces enhanced cytotoxicity with cisplatin. MTT assay to show the combined effect of antitumor drug and GLDC-shAON to the viability of A549 and H226 cells. 2,000 cells were seeded into 96-well microplate for overnight incubation followed by doxorubicin/cisplatin and shAON treatments for 3 days. The absorbance was normalized to the vehicle control. The data represent mean ± SEM of three independent experiments (**p < 0.01).

In most cases, treatment for lung cancer uses combination of two chemotherapeutic agents to achieve higher efficacy because each drug works by different mechanisms. The combination of GLDC-shAON and cisplatin enhanced cytotoxicity in A549 and H226 cells, but this was not observed with doxorubicin (Figure 6). To explore the therapeutic potential of GLDC-shAON in other types of cancer, we treated PC-3 prostate cancer cells, HeLa cervical cancer cells, U-87MG brain cancer cells, and Hep3B liver cancer cells with this compound. We found that their cell growth was significantly suppressed by GLDC-shAON treatment, with a decent IC50 of less than 30 nM (Figure S1 in Supplementary Material). Together, these findings demonstrate that GLDC inhibition is a good anticancer strategy, either works independently or combines with conventional chemotherapeutic agent.

## Discussion

In recent years, there are increasing studies explore the use of RNA-binding antisense oligonucleotides in treating different diseases including cancer, neurological disorders, and inflammatory disorders. The central idea is to reduce the protein expression of a specific gene that is associated with disease development (20). There are several strategies to interfere the function of RNA molecules, including modulating exon splicing, blocking protein binding to RNA sequences, antagonizing microRNA activities, and impairment of folding (20). However, there is a concern about the feasibility and biosafety of these compounds in human body. In a recent report, systemic administrated 2′-*O*-methoxyethyl antisense oligonucleotides are cleared *via* urine suggesting the safety of this compound in human body (21). Notably, drisapersen, an antisense oligonucleotide, has been developed into phase 3 clinical trial for Duchenne muscular dystrophy (22). In this study, shAONs were synthesized with RNA bases modified with 2′-*O*-methyl of phosphorothioate backbone (2OMePS) as described previously (17). It has been reported that shAON with 2OMePS had a mean terminal half-life of 29 days in human body (23). Therefore, repeating shAON treatment in our mouse model might result in the accumulation of GLDC-shAON in the body which in turn led to tumor suppression. Indeed, we observed GLDC exon splicing and GLDC protein reduction in GLDC-shAON-treated tumors.

Glycine decarboxylase has been reported to play a crucial role in lung tumorigenesis (6). The expression of serine–glycine biosynthetic genes including PHGDH, PSAT1 and GLDC is correlated to tumor grade and shorter disease-free survival in patients with phyllodes tumor (24). Overexpression of GLDC in 3T3 mouse fibroblast cells induces tumor formation upon inoculation into immunocompromised mice (6). On the other hand, GLDC knockdown suppresses Huh-7 liver cancer and A549 LUAD cell growth (6, 10). Cells with high SHMT2 level are sensitive to GLDC inhibition because the excess glycine that is not metabolized by GLDC can be converted into toxic metabolites that impair cell growth (7). Similarly, in this project, GLDC inhibition by GLDC-shAON is able to inhibit cell growth and tumorigenesis in both lung cancer cells and tumor xenograft. Despite significant cytotoxicity was observed in scramble-shAON, GLDC-shAON is much more potent therefore producing wider therapeutic window especially when cancer cells express high level of GLDC. Indeed, no significant cytotoxicity is reported in HLF and MRC-5 normal cells after GLDC-shAON treatment (17).

^13^C MRS has been extensively used in cancer research to study the Warburg effect in cancer cells (12). Since cancer cells upregulate the conversion of pyruvate to lactate as the results of Warburg effect (aerobic glycolysis), it is useful to measure the *in vivo* LDH flux with hyperpolarized [1-^13^C]pyruvate. Hyperpolarized [1-^13^C]pyruvate is also the only probe that has been translated into humans, in which its first-in-man was performed in prostate cancer patients in 2013 (25). Here, we utilized this technique to study the changes in tumor metabolism upon GLDC inhibition. Our MRS data showed that [1-^13^C]lactate level was significantly reduced in GLDC-shAON-treated tumors as compared with the vehicle and scramble-shAON-treated tumors. This observation was validated by the reduced extracellular acidification rate that was observed in GLDC-shAON-treated cells. These results suggest that GLDC inhibition reduces the carbon flux from pyruvate to lactate. Moreover, we found that the LDH and PDH activities in GLDC-shAON-treated tumors were significantly reduced. Similar *in vitro* results were also observed in GLDC-shAON-treated cells. However, it is not obvious at this point whether the suppression of LDH and PDH activities is due to reduction in expression or direct inhibition to these enzymes. Another study reported an increase in PKM2 activity in SHMT2 knockdown cells (7). However, we did not observe any change in PKM1 and PKM2 protein expression in GLDC-shAON-treated tumors (data not shown). Nonetheless, our results demonstrate that GLDC inhibition impairs pyruvate metabolism, thus starving cancer cells from metabolic fuels.

The TCGA database shows that GLDC upregulation is not restricted to lung cancer. It is also upregulated in several types of cancer including brain, prostate, cervical, and bladder. Hence, we also explored the therapeutic effect of GLDC-shAON in other cancers in addition to lung cancer. We found that GLDC-shAON is very potent in suppressing the cell growth of PC-3, HeLa, U-87MG, and Hep3B cells, with IC50 in the nano-molar range. Moreover, a synergistic effect is also observed when GLDC-shAON and cisplatin were treated to the cells. These results suggest the potential of anti-GLDC treatment in other cancers as well as used in the combination regimen in chemotherapy for a greater effect. In fact, there are clinical studies that explore the use of antisense drug *via* inhaled application to treat asthma (26). This opens a possibility to use antisense drug in non-invasively route of administration, perhaps in treating lung cancer. However, more evidences from studies such as pharmacodynamics and pharmacokinetics are warranted before we can develop into humans.

In summary, we demonstrate the application of hyperpolarized ^13^C MRS in probing changes in tumor metabolism upon GLDC inhibition. The use of GLDC-shAON to reduce lung cancer growth was demonstrated in both cell lines and animal model. We suggest that GLDC inhibition leads to impairment in pyruvate metabolism thus possibly hindering cancer cells from acquiring critical metabolic fuels.

## Ethics Statement

All animal procedures were approved by Institutional Animal Care and Use Committee from A*STAR Singapore.

## Author Contributions

CW: study design, performed experiments, data analysis and interpretation, and manuscript preparation. KK, WC, and XT: performed experiments and data analysis. TL: project supervision, study design, and manuscript preparation.

## Conflict of Interest Statement

The authors declare that the research was conducted in the absence of any commercial or financial relationships that could be construed as a potential conflict of interest.

## Supplementary Material

Figure S1 MTT assay to show the effect of glycine decarboxylase-steric hindrance antisense oligonucleotide (shAON) on cell proliferation in several cancer cell lines. 2,000 cells were seeded into 96-well microplate for overnight followed by shAON transfection for 3 days. The data represent mean ± SEM of three independent experiments.
