# Supplementary figures and images for: Inhibiting Glycine Decarboxylase Suppresses Pyruvate-to-Lactate Metabolism in Lung Cancer Cells
Source: Front Oncol. 2018 Jun 1;8:196. doi: 10.3389/fonc.2018.00196 (PMC5992284; doi:10.3389/fonc.2018.00196)

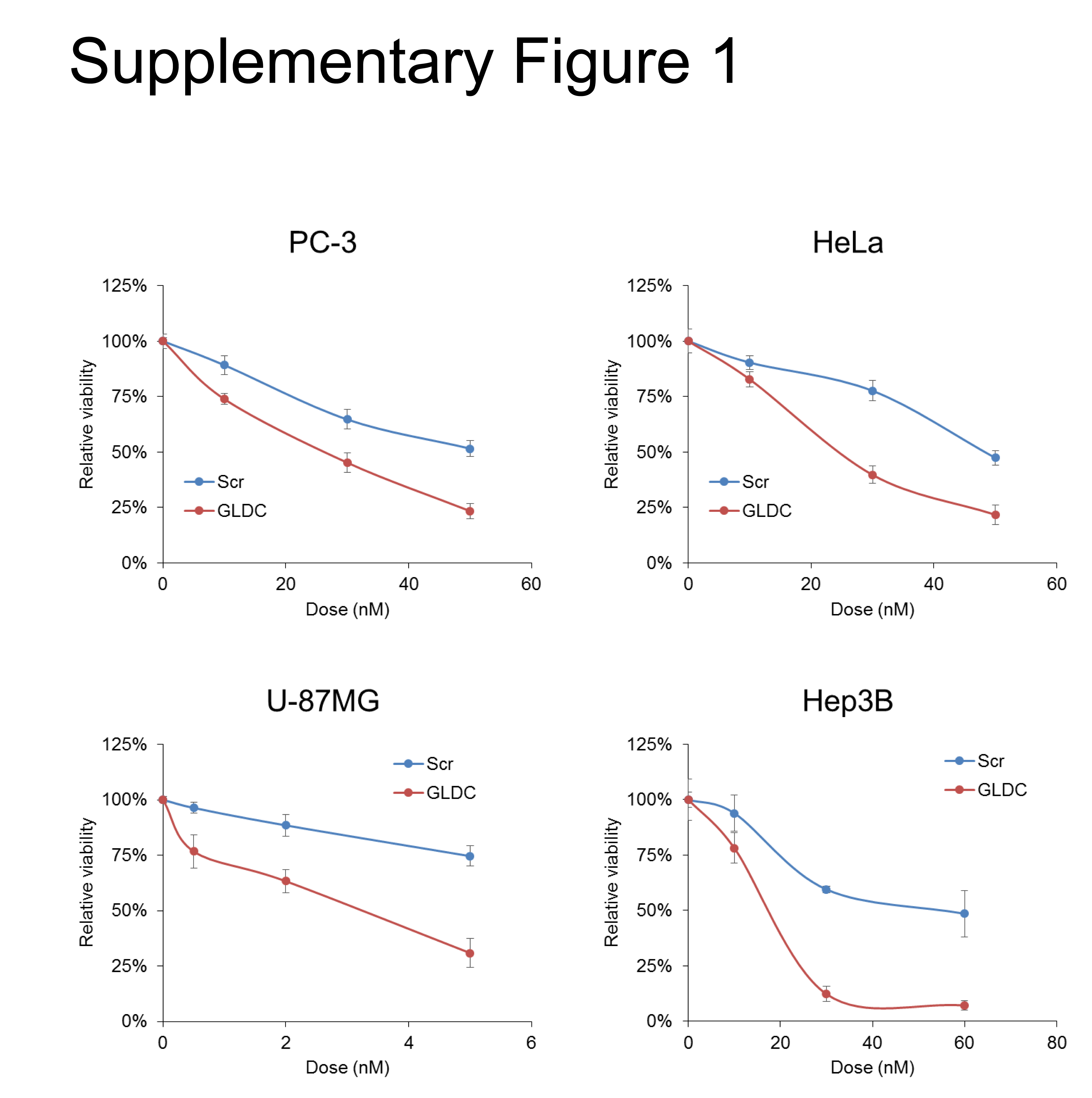

Supplement: Figure S1 — MTT assay to show the effect of glycine decarboxylase-steric hindrance antisense oligonucleotide (shAON) on cell proliferation in several cancer cell lines. 2,000 cells were seeded into 96-well microplate for overnight followed by shAON transfection for 3 days. The data represent mean ± SEM of three independent experiments. [file image_1.tif]
