# Supplementary material for: Inhibiting Glycine Decarboxylase Suppresses Pyruvate-to-Lactate Metabolism in Lung Cancer Cells
Source: Front Oncol. 2018 Jun 1;8:196. doi: 10.3389/fonc.2018.00196 (PMC5992284; doi:10.3389/fonc.2018.00196)
Supplement: Supplementary file 2 [file table_1.docx]

**Supplementary Material**

**Inhibiting glycine decarboxylase suppresses pyruvate-to-lactate metabolism in lung cancer cells**

Chern Chiuh Woo, Kavita Kaur, Wei Xin Chan, Xing Qi Teo, Teck Hock Philip Lee^*^

*Correspondence: Teck Hock Philip Lee: [philip_lee@sbic.a-star.edu.sg](mailto:philip_lee@sbic.a-star.edu.sg)

**Legends for Supplementary Figure**

Figure S1. MTT assay to show the effect of GLDC-shAON on cell proliferation in several cancer cell lines. 2000 cells were seeded into 96-well microplate for overnight followed by shAON transfection for 3 days. The data represent mean ± SEM of three independent experiments.

**Supplementary Table 1:** shAON sequences and qPCR primer sequences.

| Primers | Sequence |
| --- | --- |
| Scramble-shAON | CCUUCCCUGAAGGUUCCUCC |
| GLDC-shAON | AAGGUCAGUAGCACAGCAGGCCAGGCU |
| GLDC | F: CCAGACACGACGACTTCGC |
|  | R: CAATTCATCAATGCTCGCCAG |
| SHMT1 | F: TCCTCACATGACAAGATGCTG |
|  | R: ATTCTCCGAGGCAATCAGC |
| SHMT2 | F: CGAGTTGCGATGCTGTACTT |
|  | R: CTGCGTTGCTGTGCTGAG |
| TBP | F: ACTCCACTGTATCCCTCCCC |
|  | R: TATATTCGGCGTTTCGGGCA |
| GLDC exon skipping | F: TGTTCCAGTACCCAGACACG |
|  | R: CTGCTCGCTTGAGACCTTCT |

**Supplementary Table 2:** List of antibodies.

| **Antibodies** | **Source** | **Cat no.** |
| --- | --- | --- |
| GLDC | Cell Signaling | 12794 |
| SHMT1 | Cell Signaling | 80715 |
| SHMT2 | Santa cruz | sc-25064 |
| β-actin | Cell Signaling | 4970 |
| Goat anti-Mouse | LI-COR Biosciences | 925-32210 |
| Goat anti-Rabbit | LI-COR Biosciences | 925-32211 |
| Donkey anti-Goat | LI-COR Biosciences | 925-32214 |

**Supplementary Table 3:** Fold change of tumor versus normal samples of RNAseq data in different types of carcinoma

| **Disease types** | **Fold change versus normal samples** |
| --- | --- |
| Cervical Squamous Cell Carcinoma and Endocervical Adenocarcinoma (CESC) | 4.60 |
| Glioblastoma Multiforme (GBM) | 2.02 |
| Lung Adenocarcinoma (LUAD) | 2.39 |
| Lung Squamous Cell Carcinoma (LUSC) | 1.59 |
| Prostate Adenocarcinoma (PRAD) | 3.81 |
| Bladder Urothelial Carcinoma (BLCA) | 4.42 |

Broad Institute TCGA Genome Data Analysis Center (2017): Firehose Version 1.1.36 run. Broad Institute of MIT and Harvard.
